# MYB Transcription Factors and Its Regulation in Secondary Cell Wall Formation and Lignin Biosynthesis during Xylem Development

**DOI:** 10.3390/ijms22073560

**Published:** 2021-03-30

**Authors:** Ruixue Xiao, Chong Zhang, Xiaorui Guo, Hui Li, Hai Lu

**Affiliations:** 1 Beijing Advanced Innovation Center for Tree Breeding by Molecular Design, Beijing Forestry University, Beijing 100083, China; rxxiao1618@163.com (R.X.); 830lihui@163.com (H.L.); 2 College of Biological Sciences and Biotechnology, Beijing Forestry University, Beijing 100083, China; chongzhang@bjfu.edu (C.Z.); guoxiaorui0404@163.com (X.G.)

**Keywords:** secondary cell wall biosynthesis, MYB transcription factors, lignification, classification, MYB46/83

## Abstract

The secondary wall is the main part of wood and is composed of cellulose, xylan, lignin, and small amounts of structural proteins and enzymes. Lignin molecules can interact directly or indirectly with cellulose, xylan and other polysaccharide molecules in the cell wall, increasing the mechanical strength and hydrophobicity of plant cells and tissues and facilitating the long-distance transportation of water in plants. MYBs (v-myb avian myeloblastosis viral oncogene homolog) belong to one of the largest superfamilies of transcription factors, the members of which regulate secondary cell-wall formation by promoting/inhibiting the biosynthesis of lignin, cellulose, and xylan. Among them, MYB46 and MYB83, which comprise the second layer of the main switch of secondary cell-wall biosynthesis, coordinate upstream and downstream secondary wall synthesis-related transcription factors. In addition, MYB transcription factors other than MYB46/83, as well as noncoding RNAs, hormones, and other factors, interact with one another to regulate the biosynthesis of the secondary wall. Here, we discuss the biosynthesis of secondary wall, classification and functions of MYB transcription factors and their regulation of lignin polymerization and secondary cell-wall formation during wood formation.

## 1. Introduction

The thickening of the secondary cell wall (SCW)—that is, its lignification, is crucial in the development of secondary xylem, and its structure determines the characteristics of plant cells and organ development [1,2]. Cell wall formation of xylem cells involves the synthesis and deposition of secondary wall components, including cellulose, xylan, cell wall proteins and lignin [3]. After the lignin monomer is synthesized in the cytoplasm or near the endoplasmic reticulum, it passes through the cell membrane from the synthesis site into the developing cell wall through a series of transport mechanisms and promotes biosynthesis of the secondary wall [4]. Therefore, it is important to analyze the mechanism of SCW formation to improve wood properties and yield.

Numerous transcription factors (TFs) involved in SCW formation have been identified by gene editing and transgenic technologies in the model plant *Arabidopsis* (*Arabidopsis thaliana*) [5,6]. TFs are classified according to the structure of their DNA-binding domain, such as bZIP (basic region-leucine zipper), bHLH (basic helix-loop-helix), NAC [NAM (No apical meristem), ATAF1 (*Arabidopsis* transcription activation factor 1), ATAF2, CUC2 (Cup-shaped cotyledon 2)], MYB (v-myb avian myeloblastosis viral oncogene homolog), AP-1 (activator protien-1), WRKY (named because of its special heptapeptide conservative sequence WRKYGOK), TCP [TEOSINTE BRANCHED 1 (TB1), CYCLOIDEA (CYC) and PROLIFERATING CELL FACTORS (PCFs)], and AP2/ERF (APETALA2/ethylene-responsive factor). In plants, the MYB superfamily is one of the most abundant classes of TFs and is indispensable for SCW biosynthesis [7]. It has been a hotspot in the study of the plant transcription factor’s function because of its large number of genes, functions and different types [8]. MYB transcription factors are involved in regulating almost all aspects of plant growth, development and metabolism during the whole of the plant’s life. They mainly regulate plant responses to biotic and abiotic stresses, cell proliferation and differentiation, histomorphogenesis, organ formation and the contents and types of primary and secondary metabolites of plant metabolic pathways [7,8,9,10,11,12,13,14]. Here we review MYB-mediated SCW formation with emphasis on recent insights into this process, highlighting new concepts and areas that remain to be explored.

This review is divided into three sections—in the first section, we discuss the mechanism of SCW biosynthesis—wood formation, lignin production, and deposition. In the second section, we discuss MYB TFs in plants—their classification and roles in SCW production. Finally, we discuss the importance of MYB TFs for SCW formation during wood formation.

## 2. Mechanism of Secondary Wall Biosynthesis

### 2.1. Wood Formation

Wood is produced by the activity of the vascular cambium, and requires a complex developmental program involving the proliferation of vascular cambium cells, xylem cell differentiation and expansion, formation of the SCW, lignification and programmed cell death and, finally, mature secondary xylem (including xylem parenchyma cells, vessel, tracheary elements and et al.) formation [15,16]. The specific procedures for wood formation are as follows.

The earliest (primary) meristems are of embryonic origin. These meristems produce the primary plant body, including the primary vasculature. Meristematic cells are small, cytoplasmic, and undifferentiated. As these cells divide, the outermost cells are pushed away from the meristem, where they cease division, initiate turgor-driven cell expansion, and differentiate into specialized cell types [17]. The growth of secondary xylem depends on the division of cells in the vascular cambium. Genome-wide expression profiling of the xylem and phloem formation layers in *Arabidopsis* root hypocotyls indicates that the G2-like, NAC, AP2, MADS (MCMl, AGAMOUS, DEFICIENS, SRF), and MYB TF families play important roles in xylem and phloem cell differentiation and activation [18]. In the final stage of wood development, the tracheary elements and fibrocytes undergo programmed cell death. This is accompanied by the degradation of protoplasts and some unlignified secondary walls [19].

**Figure 1:**
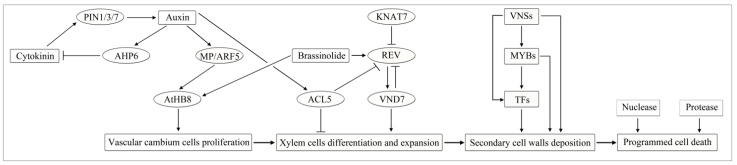
Developmental stages of wood formation and characteristic features of each stage. Note: PIN1, auxin eux carrier PIN-FORMED1 protein; MP/ARF5, the auxin-responsive transcription factor (TF) AUXIN RESPONSE FACTOR 5/MONOPTEROS; AtHB8, ARABIDOPSIS THALIANA HOMEOBOX 8; AHP6, an auxin that positively regulates the expression of an inhibitor of CK signaling; RVE, one of five HD-ZIP III genes in *Arabidopsis thaliana*; KNAT7, KNOTTED-LIKE HOMEOBOX OF ARABIDOPSIS THALIANA 7; ACAULIS5 (ACL5), encodes a thermospermine synthase. Solid arrows and flat-headed arrows represent positive and negative transcriptional regulation, respectively. Referred to [23,24,25,26].

Lignification is the final step of xylem cell differentiation. Lignification refers to a biological process in which lignin formed by the oxidative polymerization of lignin monomers. Lignin is mainly deposited on the cell walls of the tracheal components and fibers of plants, after the end of radial growth of xylem cells. This process is divided into cell-autonomous lignification and non-cell-autonomous lignification [20,21]. In cell-autonomous lignification, lignin monomers are produced and deposited in the differentiated cells. Whereas in non-cell-autonomous lignification, lignin monomers are produced by adjacent non-lignified cells, transported, and deposited on the cell wall. Using histochemistry and fluorescence microscopy techniques to study lignin deposition in the three-layer structure (S1, S2, and S3) in the secondary xylem cell wall during poplar development, it was found that there are three stages of lignification. First, lignin is deposited in the primary corner and mesial layer. Second, microfibrils continue to polymerize with the participation of pectin and other factors to form the S1 and S2 layers and lignin begins to extend from the corner to the secondary wall multilayer structure and other regions of the intercellular layer. Third, when S3 begins to form, lignin is rapidly deposited on the cell wall [22]. Wood at maturity is essentially the remains of secondary walls, so understanding the biosynthesis of secondary wall components can be used as a genetic tool to develop wood (Figure 1).

### 2.2. Lignin Production and Deposition

Lignin production and deposition comprise lignin monomer synthesis in the cytoplasm, lignin monomer transport through the cell membrane, and lignin monomer oxidative polymerization on the cell wall [27]. The following is our detailed introduction to the three processes.

#### 2.2.1. Biosynthesis of Lignin Monomers

In most plants, the biosynthesis of lignin polymers occurs primarily via the phenylpropane pathway and the lignin-specific pathway. The multiple-branch pathways of the phenylpropane pathway generate a variety of compounds that, for example, provide structural support and increase cell and stem strength and bending resistance [28,29]. A series of hydroxylation, methylation and reduction reactions occurs in the phenylalanine metabolic pathway, which involves a series of continuous enzymatic reactions. The genes encoding these single-lignol biosynthetic enzymes have been identified and their functions have been characterized in many species [30]. The phenylalanine metabolic pathway comprises three major steps. The first is the phenylpropane pathway, which includes phenylalanine ammonia lyase (PAL), cinnamate 4-hydroxylase (C4H), and 4-coumarate-CoA ligase (4CL). The phenylpropane lignin-synthesis pathway starts with the generation of cinnamic acid by PAL-phenylalanine. C4H is one of the best-characterized cytochrome P450 hydroxylases in higher plants and catalyzes the conversion of cinnamic acid to p-coumaric acid. Next, p-coumaric acid is activated to 4-coumaroyl-coenzyme A (CoA) by 4CL. Also, 4CL catalyzes the conversion of ferulic acid and erucic acid into ferulyl-CoA and erucyl-CoA. However, the formation of ferulic acid and erucic acid is based on a one-step monomer-methylation reaction involving caffeine-O-methyltransferase (COMT) and ferulic acid 5-hydroxylase (F5H). The third step is monomer synthesis, which involves cinnamoyl-CoA reductase (CCR) and cinnamyl alcohol dehydrogenase (CAD) in the formation of the monomers p-coumarol, coniferyl alcohol, and erucyl alcohol. The three monomers are converted to p-hydroxyphenyl lignin, guaiacyl lignin and syringyl lignin by laccase (LAC) and peroxidase (POX) [31]. MYB TFs play an important role in the biosynthesis of lignin. For example, PAL (MYB8, [32]; MYB15, [33]; MYB46, [34]; MYB58 and MYB63, [35]), C4H (MYB15, [33]; MYB46, [34]), 4CL (MYB15, [33]; MYB46, [34]; MYB58 and MYB63, [35]), HCT (MYB46, [34]), C3H (MYB46, [34]; MYB58 and MYB63, [35]), CCoAOMT (MYB46, [34]; MYB58 and MYB63, [35]), F5H (MYB103, [35,36,37]), CCR (MYB46, [34]; MYB58 and MYB63, [35]), and CAD (MYB15, [33]; MYB46, [34]; MYB58 and MYB63, [35]).

As a model plant, *Arabidopsis* has advantages that other plants cannot replace, such as: short growth cycle; small genome; only five chromosomes; with all the characteristics of dicot plants; effective Agrobacterium-mediated transformation pathway, easy to obtain a large number of mutants and genome resources; small size, can be planted in large quantities and more seeds. Analyzing the regulatory network of MYB transcription factors in the formation of the *Arabidopsis* secondary wall can promote related research in other plants. A large number of studies have also proved that *Arabidopsis* is a good material for studying the formation of secondary walls [5,6,33,35,37]. Therefore, we take *Arabidopsis* as an example and draw Figure 1 to help you understand the important role of MYB transcription factor in the process of lignin synthesis more intuitively.

#### 2.2.2. Transportation of Lignin Monomers

Lignin-monomer synthesis occurs in the cytoplasm or near the endoplasmic reticulum, rather than on the cell wall [38]. Therefore, lignin monomers cross the cell membrane from the site of cytoplasmic synthesis and enter the developing cell wall, which involves a series of transport mechanisms and may occur by passive diffusion, vesicle-mediated extracellular secretion, or ATP-dependent transport by ABC transporters or proton-coupled antitransporters [39,40,41]. The latter mechanism is merely hypothetical at present. *ABCG11*, *ABCG22*, *ABCG29*, and *ABCG36* can be co-expressed with *MYB58* in the differentiated *Arabidopsis* tubular molecular cell culture system to jointly regulate the expression of lignin-monomer-synthesis genes [42]. Finally, the lignin monomers are transported as single lignin glycosides by UDP-glucosyltransferase. The mechanism by which lignin monomers are transported to the lignification site in the cell wall is unclear, and further research is needed.

#### 2.2.3. Oxidative Polymerization and Deposition of Lignin Monomers

**Figure 2:**
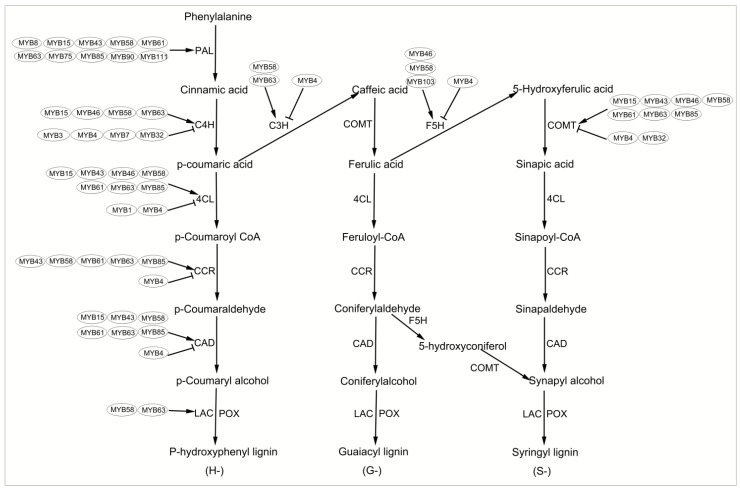
Phenylpropane lignin biosynthesis pathway in *Arabidopsis*. In this model, MYB transcription factors control the expression of genes in the lignin synthesis pathway. MYB58 and MYB63, MYB4 can activate/inhibit almost all the enzymes in the lignin synthesis pathway, respectively. All TFs appear to be in ovals in the figure. Solid arrows and flat-headed arrows represent positive and negative transcriptional regulation between transcription factors and enzymes, respectively. The other solid arrows represent the direction of the regulatory network. Note: PAL, phenylalanine ammonia lyase; 4CL, 4-coumarate-CoA ligase; C4H, cinnamate 4-hydroxylase; CCR, cinnamyl-CoA reductase; CAD, cinnamyl alcohol dehydrogenase; C3H, p-coumaric acid 3-hydroxylase; COMT, catechol-O-methyltransferase; F5H, ferulic acid 5-hydroxylase; POX, peroxidase; LAC, laccase.

The lignin monomer moves freely in the cell, and the immobilized oxidase stops its movement and determines the position of lignin polymer formation. LAC and type III POX catalyze the oxidative polymerization of lignin monomers. POX and LAC undertake the oxidative polymerization of monolignol to the following lignin polymers: p-hydroxybenzene lignin (H monolignol), guaiac lignin (G monolignol), and syringyl lignin (S monolignol) (Figure 2) [43]. Monolignol polymerization is in part non-cell autonomous and occurs mainly after programmed cell death [44]. An *Arabidopsis* double mutant revealed that LAC is involved in the biosynthesis of lignin. Among the 17 known genes in the LAC family, eight are expressed in the stem and four (*LAC4*, *LAC11*, *LAC15*, and *LAC17*) play a role in lignin biosynthesis [45]. The lignin level in the seed coat of the LAC15-deficient *tt10 Arabidopsis* mutant was 30% lower than that of the wild type [46]. Other studies have confirmed that co-expression of LAC4 and LAC17 with the *CesA* gene [47] and the CAD genes *CAD-C* and *CAD-D* is involved in the biosynthesis of monoxyphenol [48]. Zhou et al. [35] determined that MYB58 and MYB63 are transcriptional activators of lignin biosynthesis, and MYB58 directly activates LAC4. A *lac11* single-knockout mutant exhibited normal lignin deposition, but *lac11 lac4* and *lac11 lac17* double-knockout mutants exhibited only a slight reduction in lignin level. However, simultaneous disruption of LAC4, LAC11, and LAC17 almost completely eliminated lignin deposits, resulting in severe damage. SND1 and MYB46 are transcriptional activators of the LAC11 promoter, and MYB58 is a less efficient activator of the LAC11 promoter (Figure 2) [49]. After LAC initiates oxidative polymerization, POX forms rigid cross-links between lignin, hemicellulose and extensin, which in turn affects lignification [50]. Single or double protrusion of AtPRX2, AtPRX71, and AtPRX25 reduces the accumulation of lignin without affecting plant height [51,52]. The lignification of xylem tubular molecules and fibers depends on AtLAC4, AtLAC11, and AtLAC17 [45]. Therefore, LAC and POX independently and interdependently affect lignified tissues.

## 3. MYB Transcription Factors in Plants

### 3.1. The Classification of MYBs

The first MYB gene identified in plants was *C1* from maize, which is involved in anthocyanin biosynthesis [53]. Since then, an increasing number of MYB genes has been identified and characterized in numerous plant species [54]. MYB TFs are implicated in regulating SCW biosynthesis genes directly in plants. MYB proteins have two distinct regions, an N-terminal conserved MYB DNA-binding domain and a diverse C-terminal modulator region responsible for regulatory activity. Based on the number of MYB domains at the N terminus, the MYB family is divided into R1-MYB (1R-MYB), R2R3-MYB (2R-MYB), and R1R2R3-MYB (3R-MYB) [55]. TFs in the smallest class, R0R1R2R3-MYB (4R-MYB), whose members have four R1/R2-like repeats, have also been found in plants such as *Arabidopsis* [56] and soybean [*Glycine max* (Linn.) Merr.] [57]. In plants, the first tryptophan of R3 is substituted for phenylalanine or isoleucine.

### 3.2. Functions of the Categories of MYBs

All four MYB classes are found in plants, which have the highest diversity of MYB proteins. The 1R (R1-type MYB) proteins contain a unique MYB-binding domain, spanning 53 amino-acid residues including three equidistant tryptophans that may form an HTH structure for DNA recognition [58]. The R1-MYB class is fairly divergent and include factors that bind the consensus sequence of plant telomeric DNA (TTTAGGG) [59]. Members of R1-MYB are responsible for cellular morphogenesis, secondary metabolism, organ morphogenesis, phosphate starvation, chloroplast development, and circadian regulation in plants [13].

Most of the lignin TFs in the R2R3-MYB family are unique to plants [60]. In contrast to the highly conserved MYB domain, the other regions of R2R3-MYB proteins are highly variable. Based on the conservation of the DNA-binding domain and the amino-acid motifs in the C-terminal domain, R2R3-MYB members are divided into at least 25 subgroups in *Arabidopsis*, of which the members of each have similar or identical functions [14,61]. For instance, some R2R3-MYB proteins in subgroup 3 (Sg3) (AtMYB58, AtMYB63, and AtMYB85) and subgroup 21 (Sg21) (AtMYB52, AtMYB54, and AtMYB69) positively regulate lignin biosynthesis in the cell wall [35]. Members of subgroup 4 (Sg4) (AtMYB3, AtMYB4, AtMYB7, and AtMYB32) act as repressors of the lignin-biosynthesis pathway [60,62]. The functions of each R2R3-MYB subgroup in plants are shown in Table 1.

The R1R2R3-type MYB (3R-MYB) proteins are typically encoded by five genes in higher plant genomes [13] and regulate the transcription of cyclin genes via MYB recognition elements in cyclin promoters, thereby controlling the cell cycle [56]. Members of the 3R-MYB class also control cellular morphogenesis [84,85] and secondary metabolism [86,87], encode core components of the central circadianoscillator [88], and encode proteins involved in organ morphogenesis [89], chloroplast development [90], and the responses to phosphate starvation [91]. However, the contributions of 3R-MYB factors require further research. A single 4R-MYB protein is encoded in several plant genomes, but its function is unclear.

## 4. Regulation of MYBs in Lignification

It can be seen from the above that MYB transcription factors play a very important role in plant secondary wall biosynthesis. However, the regulation of transcription factors on plants is not single but is regulated by levels of transcription factors at different levels, forming a huge regulatory network and playing a regulatory role. So, what is the regulation between MYB transcription factor and plant secondary wall synthesis? Next, we will try to explain it in detail. In this part, we discuss first the regulation of SCW biosynthesis by MYB46/83 as the second main switch. Next, we consider how other MYB TFs regulate cell-wall biosynthesis in plants.

### 4.1. Mechanism by Which MYBs Regulate Lignification

MYB transcription activators/repressors participate in various enzymatic reactions in the phenylpropane metabolic pathway to regulate lignification (Figure 2) [92,93]. Detailed promoter and electrophoretic mobility shift assay of phenylpropane biosynthetic genes, including *PAL* and *4CL*, has shown that the cis-elements corresponding to the MYB TF-binding motif are necessary for coordinated activation of monolignol pathway genes [35,94,95,96,97]. One such element is the AC element (also known as C1-motif, PAL-box, or H-box, divided into I, ACCTACC; II, ACCAACC; and III, ACCTAAC), which is rich in AC sequences. With few exceptions, MYB TFs regulate gene expression by binding to AC elements in the promoter regions of downstream lignin biosynthesis-pathway genes [26,98]. When MYB combines with a specific promoter, the second and third helices form an HTH structure and the third helix functions to directly recognize a particular DNA sequence motif [14]. In SCW biosynthesis, in-depth exploration of the binding mode between MYB transcription factors and AC elements will enable editing of AC elements by genetic engineering to regulate SCW synthesis. However, the mechanisms underlying the selective binding of SCW TFs to the promoters of specific SCW-biosynthesis genes are unclear.

### 4.2. MYB46 and MYB83 Are the Second Layer of the Main Switch for Secondary Cell-Wall Biosynthesis

**Figure 3:**
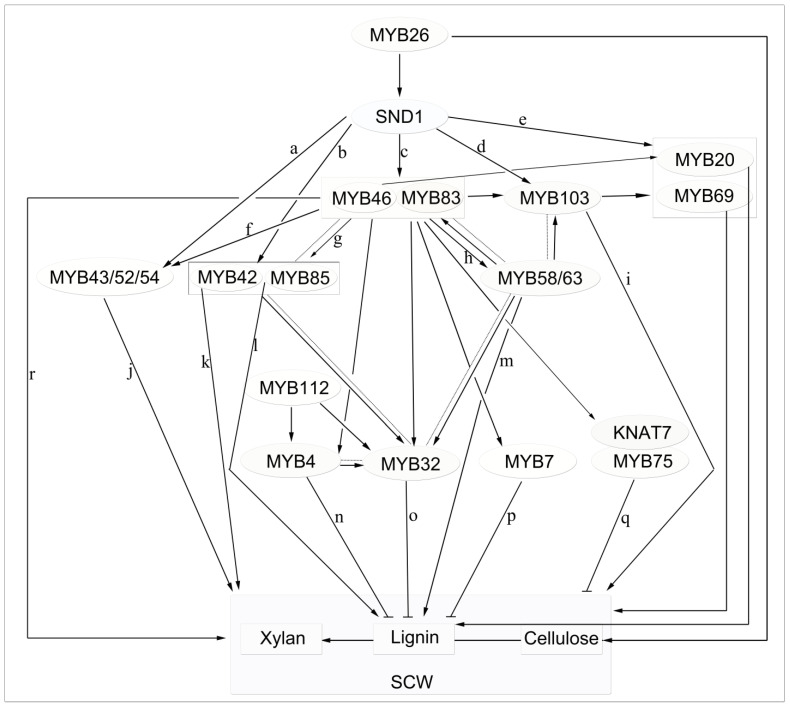
Proposed model of MYB-mediated secondary cell wall (SCW) regulation in *Arabidopsis*. Solid black and flat-headed arrows represent positive and negative transcriptional regulation, respectively. Dashed lines represent co-expression relationships, MYB26 and SND1, MYB46/83, and other TFs represent the first, second, and third layers of the transcriptional regulatory network, respectively. The expression of 9 SND1-regulated transcription factors, namely, MYB20, MYB42, MYB43, MYB52, MYB54, MYB69, MYB85, MYB103, was developmentally associated with cells undergoing secondary wall thickening (a, b, c, d, e; [72]). MYB46 and MYB83 serve as the second layer of the main switch for secondary cell wall biosynthesis, which activate downstream transcription factors (including MYB20, MYB42, MYB43 and MYB85) by binding to SMRE sequence in an SCW MYB-responsive element (f, g, h, r; [101,103]) and directly or indirectly regulate the biosynthesis of the secondary wall. MYB42, MYB43, MYB85 (j, k, l; [109]), MYB58, MYB63 (m; [110]), MYB103 (i; [37]) are transcriptional regulators that directly activate lignin biosynthesis genes during secondary wall formation in *Arabidopsis*. MYB4, MYB7, MYB32, MYB75 are inhibitors of lignin biosynthesis (n, o, p, q; [60]). The concerted actions of the MYB TFs in this network leads to a coordinated activation of SCW biosynthetic genes, which results in the synthesis of lignin, cellulose, xylan.

MYB TFs can be activated in multiple ways. Throughout the formation of the secondary wall, the NAC (NAM/ATAF/CUC) TFs acts as the first-level main switch of SCW biosynthesis and activates downstream TFs to regulate the entire SCW biosynthetic network. MYB46/MYB83 act as the second-level main switch of SCW biosynthesis, serving as molecular tools for improving plant biomass (Figure 3) [26,99].

MYB46 and MYB83—two of the earliest discovered lignin-specific TFs—are direct targets of SND1 (secondary wall-associated NAC domain) in *Arabidopsis* and not only modulate the lignin synthesis pathway but also redundantly activate SCW formation [35,71,72,100]. MYB46 and MYB83 are expressed in vascular tissue and fibers, and their dominant inhibition or RNA interference inhibition markedly suppresses secondary-wall thickening in fibers and vascular tissue leading to collapse of the vascular phenotype. Similar to secondary wall NAC (SWN), overexpression of MYB46 and MYB83 induced ectopic secondary cell wall synthesis [72,100,101]. By analyzing the promoter sequences of downstream genes regulated by MYB46, Zhong et al. found that MYB46 and MYB83 regulate SCW biosynthesis during wood formation by binding to a 7-bp conservative DNA sequence in an SCW MYB-responsive element [SMRE, Secondary wall MYB-responsive element; ACC(A/T)A(A/C)(T/C)] [102,103]. However, the regulation of SCW biosynthesis is more complex than formerly thought. The expression of SCW-biosynthesis genes is regulated by the coordinated actions of multiple MYBs, including activators and repressors [35,104], via binding to not only AC elements (one type of SMRE) but also other SMRE sites. Similar to the promoters of lignin-biosynthesis genes, those of cellulose- and xylan-biosynthesis genes contain multiple SMRE sequences, suggesting that MYBs bind to and activate SMRE sites in the promoters of cellulose- and xylan-biosynthesis genes. Another MYB TF, MYB26, may act as a master switch of SCW biosynthesis in anther endothelial cells—its mutation causes the loss of anther endothelial cell secondary-wall thickening and the anther-dehiscence phenotype. Also, its overexpression leads to ectopic deposition of the secondary wall [105]. MYB26 directly regulates NST1 and NST2, which are critical for inducing secondary thickening biosynthesis genes [106]. The four functional homologous genes MYB TF (PtrMYB2/3/20/21) of MYB46/83 in another model plant poplar, PtrMYB2/3/20/21, are also transcriptional master switches controlling secondary-wall biosynthesis during wood formation that bind to secondary wall NAC-binding element (SNBE) sites in their target gene promoters, thereby activating their expression [107]. Interestingly, the four PtrMYBs exhibit marked differences in how they activate their target genes. One possibility is that they exhibit differential expression patterns in different organs and tissues [108]. Alternatively, they may have different binding affinities for the various SMRE sequences in the promoters of their target genes.

The discovery of the hierarchical transcriptional network that regulates SCW biosynthesis in *Arabidopsis* was a major breakthrough. However, the regulation of secondary wall formation is more complex than formerly thought, involving positive and negative regulation, dual function regulation, feedback loops, and crosstalk among combinatorial complexes and pathways [103]. Does this affect the transmission of signals related to lignin synthesis by influencing TF-TF, MYB gene-TF, and/or MYB gene-MYB gene interactions? Clarification of the SCW regulatory network warrants further research.

### 4.3. Downstream Targets of MYB46/MYB83

#### 4.3.1. In *Arabidopsis*

MYB46 and MYB83 activate downstream TF expression [105]. From the metabolic model, MYB46 and MYB83 regulate a series of downstream MYB TFs involved in lignin biosynthesis, including the lignin-activating factors MYB58, MYB63, and MYB85 and the lignin inhibitors MYB4, MYB7, and MYB32 (Figure 3).

Lignin-specific MYBs—MYB58, MYB63, and MYB85—regulate the biosynthesis of lignin rather than cause the deposition of cellulose and hemicellulose (Table 2). Their overexpression leads to activation of lignin-biosynthesis genes and ectopic deposition of lignin in cells that are usually not lignified [35,72,101]. It has long been thought that lignin-specific MYBs bind to AC elements in the promoters of lignin-biosynthesis genes and thereby activate the lignin-biosynthesis pathway [36,43]. MYB58 and MYB63 were first reported as lignin-specific transcriptional activators in *Arabidopsis* [35]. They have been shown to bind to AC elements and regulate genes involved in lignin biosynthesis (including early genes such as *PAL*, *C4H*, and *4CL*) but not those involved in cellulose or xylan biosynthesis, which is congruent with the proposed model of regulation of lignin gene expression via AC cis-elements [35]. MYB85 activated the expression of the lignin-biosynthesis gene *4CL1* in a transient assay of *Arabidopsis* protoplasts (Figure 2) [76].

MYB46, MYB83, and the downstream lignin regulator MYB4 and its homologs MYB7 and MYB32, which belong to subgroup 4 of R2R3-MYB TFs, directly inhibit lignin biosynthesis [62,111,126,127]. The promoter element bound by MYB4 [the 7-bp conserved sequence ACC(A/T)A(A/C)(T/C)] is similar to the SMRE of *Arabidopsis*. MYB4 regulates the expression of genes related to SCW synthesis by binding to the SMRE sites of downstream target genes or via mitogen-activated protein kinase in *A. thaliana* and *Pinus taeda* [26,105]. MYB4, MYB7, and MYB32 have a conserved ethylene-reactive element binding factor-related amphiphilic repression (EAR) motif and GY/FDFLGL motif at the C terminus [62,111]. The GY/FDFLGL motif contributes to the interaction between MYB TFs and SUPER SENSITIVE TO ABA AND DROUGHT 2 (SAD2) [111]. SAD2 is an imported β-like protein that mediates the nuclear translocation of MYB4, MYB7, and MYB32 as well as inhibits the expression of its target genes (e.g., *C4H*) (Figure 2) [111]. MYB3 is a newly discovered repressor of phenylpropane biosynthesis in *A. thaliana* and is one of the four members of R2R3-MYB subgroup 4 [62]. The inhibition by MYB3 of C4H expression is directly regulated by the core inhibitors LNK1 and LNK2, which promote the binding of MYB3 to the C4H promoter (Figure 2) [62]. In addition, MYB repressors downregulate AtNST3/SND1 expression in vitro, and AtNST3/SND1 directly regulates AtMYB32 [93]. In view of this, negative feedback of the VNS-MYB network enables fine-tuning of the formation of secondary walls [128]. Except Sg4, members of other subgroups of MYB negatively regulate SCW biosynthesis by interacting with other TFs. For example, the MYB-R3 domain of MYB75 [114] (also known as PAP1) in *Arabidopsis* and MYB6 [116], MYB26 [106] in transgenic poplar physically interact with the KNOX TF KNAT7, forming a complex that inhibits the development of SCWs in poplar and *Arabidopsis*. The complex triggers a reduction in deposition and biosynthesis gene expression, which hinders SCW development.

#### 4.3.2. In Poplar

Most of our understanding of secondary growth comes from the study of *Arabidopsis* [129]. However, secondary growth in woody perennials is different from that in *Arabidopsis* roots or hypocotyls [130]. Therefore, identifying the genes that regulate secondary growth in representative woody plant poplar is a top priority [115]. PtrMYB2, PtrMYB3, PtrMYB20, and PtrMYB21 are the functional orthologs of *Arabidopsis* MYB46 and MYB83, and they regulate poplar secondary-wall biosynthesis by binding to and activating SMRE sequences [105,115]. Like the *Arabidopsis* SWNs [131,132], PtrWNDs bind to the SNBE sites in the promoters of PtrMYB2/3/20/21 and thereby activate their expression [107]. The findings that these four PtrMYBs all are capable of activating secondary wall biosynthetic genes in poplar trees indicate that these PtrMYBs might function redundantly in regulating secondary wall biosynthesis during wood formation. But why poplar evolved to retain all these four PtrMYBs. One possibility is that although they are all transcriptional activators of secondary wall biosynthesis, they exhibit differential expression patterns in different organs and tissues [108]. Another possibility is that they might differentially activate their target genes as they show differential binding affinity toward different SMRE sequences that are present in promoters of their target genes. Therefore, the expression of these four PtrMYBs might be required for a full strength of transcriptional activation of secondary wall biosynthesis. This is the same as MYB46 and MYB83 in *Arabidopsis* as the T-DNA knockout mutation of either MYB46 or MYB83 alone does not cause an apparent reduction in secondary wall thickening [71]. Although the functions of some orthologous R2R3-MYB TFs from *Arabidopsis* and poplar appear to be conserved in regulating SCW biosynthesis, the transcriptional regulation network of SCW biosynthesis may be different in herbaceous and woody plants. Unlike *Arabidopsis* AtMYB85 which can promote the synthesis of cellulose, lignin, and hemicellulos, its homologues PtoMYB92 and PtoMYB125 can promote the accumulation of lignin but inhibit the synthesis of hemicellulose [119]. Studies have also shown that in the phylogenetic analysis, PtoMYB216 protein groups in the lignification-related R2R3-MYB clade and it is most similar to AtMYB61 from *Arabidopsis* [124]. AtMYB61 is related to the ectopic lignification of plants [70]. PtoMYB216 is related to the modification of the cell wall of poplar xylem. This may be caused by differences in species [124]. Although the internal MYB transcription factors in plants have different regulation on the secondary wall, they all follow the hierarchical regulation mode of VNSs-MYB-TFs-SCW. Perhaps this can provide a foundation for us to further study the regulation mechanism of the secondary wall.

Similar to *Arabidopsis*, MYB subgroup 4 members—downstream regulators of PtrMYB2/3/20/21—PtoMYB156 [121], PtrMYB189 [123] and PdMYB221 [125,133,134] are negative regulators of lignin biosynthesis. This is the same as transcription factors such as EgMYB1 [135], BpMYB4 [136], CmMYB8 [137], AmMYB308 [138], ZmMYB42 [139] and ZmMYB31 [104], which are also negative regulators of lignin biosynthesis. Except for PtrMYB189, all of the above-mentioned subgroup 4 members and other MYB repressors have a C-terminally conserved EAR motif, with the expression of these essential genes for repression demonstrated in vitro and *in planta* [111,112,140]. For PtrMYB189, site-directed deletion and mutagenesis of 13 amino acids (277–289, GDDYGNHGMIKKE) at the C terminus of MYB indicated the importance of this region in target inhibition [123]. Also, numerous MYB TFs enhance cell-wall properties and wood formation. For example, PtrMYB121 directly binds to and activates the promoters of genes related to lignin and cellulose synthesis, thus regulating SCW formation [117]. PtrMYB152, the homolog of the *Arabidopsis* R2R3-MYB TF AtMYB43, acts as a specific transcriptional activator of lignin biosynthesis during the formation of poplar wood. Overexpression of PtrMYB152 increased the thickness of the secondary wall in plants [120]. PtrMYB92 [119], PtrMYB18, PtrMYB74, PtrMYB75, PtrMYB121, and PtrMYB128 [131] activate the promoters of all three main wood component-biosynthesis genes. In addition, in the third layer, the PtrMYB161 TF binds to multiple sets of target genes, allowing it to act as both an activator and a repressor [141]. It directly regulates the expression of two syringyl-specific monoxylinol genes (*PtrCAld5H1* and *PtrCAld5H2*) [133,142,143] and two key SCW cellulose-synthase genes, *PtrCesA4* and *PtrCesA18* (PtrCesA8-B) [144,145].

Recent studies have shown that changes in the status of MYB transcription factors can affect the biosynthesis of lignin. For example, phosphorylation of LTF1, an MYB transcription factor in Populus, acts as a sensory switch regulating lignin biosynthesis in wood cells. When LTF1 becomes phosphorylated by PdMPK6 in response to external stimuli such as wounding, it undergoes degradation through a proteasome pathway, resulting in activation of lignification. Expression of a phosphorylation-null mutant version of LTF1 led to stable protein accumulation and persistent attenuation of lignification in wood cells [135]. Moreover, the post-translational regulation of MYB transcription factors, especially their ubiquitination regulation, is closely related to the biosynthesis of lignin. Endoplasmic reticulum-localized E2 ubiquitin-conjugating enzyme 34 (PtoUBC34) interaction with lignin repressors MYB221 and MYB156 regulates the transactivity of the transcription factors in *Populus tomentosa*. This specific interaction allows for the translocation of TFs PtoMYB221 and PtoMYB156 to the ER and reduces their repression activity in a PtoUBC34 abundance-dependent manner [146]. The above studies show the presence of a complex MYB regulatory network in poplar, similar to that in *Arabidopsis*, which regulates secondary-wall biosynthesis. Therefore, research on the MYB regulatory networks in *Arabidopsis* and poplar will enhance the understanding of secondary-wall biosynthesis.

Other aspects of the network require further study, such as the patterns of genetic interaction within the lignin-biosynthesis pathway and how the multigene-coordinated network functions in wood formation. Therefore, plants has a complex transcriptional network that regulates its SCW deposition program, as summarized in Figure 3.

### 4.4. Other Elements That Interact with MYB Transcription Factors to Regulate Secondary-Wall Biosynthesis

#### 4.4.1. Noncoding RNAs

The regulation of secondary walls by noncoding RNAs (ncRNAs), such as microRNAs (miRNAs) and long ncRNAs (lncRNAs), has been a topic of interest. Notably, miRNAs, a class of endogenous ncRNAs consisting of approximately 21–23 nucleotides, play important roles in plant development by cleaving target mRNAs with perfect or near-perfect complementarity [147,148]. The miRNA–MYB network regulates secondary-wall biosynthesis in plants [149] by modulating the activities of enzymes (e.g., CAD and POX) related to phenylpropane metabolic pathways [150]. For example, higher expression of MYBs in MIM858 (an artificial miRNA858 target mimic) lines leads to redirection of the metabolic flux towards the synthesis of flavonoids at the cost of lignin synthesis [149]. Alternatively, miRNAs post-transcriptionally regulate MYB genes related to secondary-wall formation [14,61,151,152]. Lignin biosynthesis is also regulated by coordinated networks involving TFs, miRNAs, and lncRNAs, depending on the genetic effects of the loci [153]. High-throughput RNA sequencing showed that the interaction between lncRNAs, miRNAs, and TFs (including MYBs) contribute to wood formation in *Populus. tomentosa* [154]. There are few studies on the roles of ncRNAs and MYB TFs in SCW formation. Comparison of differentially expressed miRNA (DEmiRNA) and target gene annotation between poplar and larch suggested the different functions of DEmiRNAs and divergent mechanism in wood formation between two species [155]. To increase our understanding of SCW biosynthesis in plants, these regulatory networks involving TFs, miRNAs, and lncRNAs need to be investigated.

#### 4.4.2. Plant Hormones

MYB TFs also stimulate plant hormone-mediated plant lignification [58]. For instance, growth hormone, cytokinin, brassinolide and abscisic acid regulate SCW biosynthesis by directly regulating MYB TFs in *Arabidopsis*, rice, and other plant species [156,157]. ABA has been reported to be involved in the regulation of lignin biosynthetic genes and TF regulators that respond to the lignin accumulation process in plants [158]. For example, ABA induced lignin biosynthesis by promoting the expression of *CgMYB58* and its target genes in HR, HB and KP juice sacs [159]. The latest research shows that melatonin can affect the expression of MYB transcription factor, thereby regulating the synthesis of lignin [160]. Also, certain factors combine with hormone-related elements in the MYB promoter region to regulate plant lignification. Auxin response factors (ARFs) are important regulators of lignin biosynthesis in various biological processes in plants. ARF8.4, a flowering-related spliceosome, binds to auxin-related elements in the MYB26 promoter and activates its transcription, thereby controlling interior-wall lignification [161]. Despite these advances, the key plant-hormone-related regulatory nodes in the lignin-biosynthesis pathway have not been elucidated [60]. In-depth exploration of the regulatory network involving MYB TFs and plant hormones will facilitate genetic strategies for increasing plant lignin content.

## 5. Summary and Prospects

Although the large, plant-specific MYB gene family promotes the evolution of plant-specific physiological or developmental processes, their roles in the SCW biosynthesis regulatory network are unclear. Are other MYB TFs involved in lignin polymerization and SCW deposition? Are there differences in lignin polymerization mediated by different MYB TFs? Is there a cascade activation relationship among these MYB TFs? What is the regulatory relationship among these MYB TFs? Also, experimental verification of the regulatory network of MYB TFs is needed. Related TFs should be investigated using emerging techniques (e.g., transcriptome sequencing), and mutants could be used to determine upstream and downstream relationships in the MYB TF regulatory network. In addition, the elucidation of functional relationships between specific target genes and MYB TFs would enhance the understanding of the roles MYB-type TFs play in gene regulation in plants and promote the development of new varieties by metabolic engineering.

SCW biosynthesis involves external factors such as light and temperature, and internal factors such as TFs, enzymes, and endogenous hormones (reviewed by [162,163]). Therefore, we need to consider the impact of these factors on wood formation to be able to artificially design wood development to meet the needs of modern production and lifestyles. Although there have been major advances in our understanding of the regulation of lignin polymerization and secondary-wall formation in recent years, the roles of MYB TFs, which garnered the attention of scientists nearly 100 years ago, will continue to capture the interest of plant biologists.

## Figures and Tables

**Table 1:** Major R2R3-MYB subgroups in *Arabidopsis* and their functions.

Subgroup	Function	Representative Factors	References
Sg1/11/17/20/23	Responses to stress	AtMYB30, AtMYB60, AtMYB96, AtMYB102, et al.	[14,63]
Sg2/5/6/7/8/10/12/14	Cell patterning or secondary metabolites biosynthesis	AtMYB11, AtMYB12, AtMYB13, AtMYB15, AtMYB28, AtMYB29, AtMYB37, AtMYB38, AtMYB51, AtMYB68, AtMYB75, AtMYB76, AtMYB84, AtMYB90, AtMYB111, AtMYB113, AtMYB114, AtMYB122, AtMYB123, et al.	[64,65,66,67,68,69]
Sg3/4/13/21	Promote/Inhibit lignin, cellulose, and/or xylan biosynthesis	AtMYB3, AtMYB4, AtMYB7, AtMYB32, AtMYB46, AtMYB52, AtMYB54, AtMYB56, AtMYB58, AtMYB61, AtMYB63, AtMYB68, AtMYB69, AtMYB83, AtMYB85, AtMYB103, AtMYB105, et al.	[35,37,62,63,68,70,71,72]
Sg9/15/22/25	Control cell fate and identity	AtMYB23, AtMYB44, AtMYB66, AtMYB77, AtMYB106, AtMYB115, AtMYB118, et al.	[73,74,75,76,77,78,79]
Sg16/18/19/24	Plant development	AtMYB18, AtMYB21, AtMYB24, AtMYB33, AtMYB38, AtMYB65, AtMYB93, AtMYB101, et al.	[65,80,81,82,83]

**Table 2:** The main MYB transcription factor that regulates secondary wall synthesis in *Arabidopsis* and poplar.

Species	MYB TFs	Ortholog in *Arabidopsis thaliana*	Annotation	References
*Arabidopsis thaliana*	AtMYB3		inhibit the accumulation of lignin	[111]
	AtMYB4	-	inhibit the accumulation of lignin	[111]
	AtMYB7	-	inhibit the accumulation of lignin	[111]
	AtMYB15	-	promote the synthesis of lignin	[33]
	AtMYB20	-	promotes the accumulation of lignin	[112]
	AtMYB32	-	inhibit the accumulation of lignin	[111]
	AtMYB43	-	promotes the accumulation of lignin	[112]
	AtMYB46	-	promote the synthesis of cellulose, lignin, and hemicellulos	[113]
	AtMYBB58		promotes the accumulation of lignin	[35]
	AtMYB61	-	promotes the accumulation of lignin	[70]
	AtMYB63	-	promotes the accumulation of lignin	[35]
	AtMYB75	-	inhibit the accumulation of lignin	[114]
	AtMYB83	-	promote the synthesis of cellulose, lignin, and hemicellulos	[113]
	AtMYB85	-	promotes the accumulation of lignin	[72]
	AtMYB103	-	promotes the accumulation of lignin and cellulose	[37]
Poplar	PtrMYB2/3/20/21	MYB46/83	promote the synthesis of cellulose, lignin, and hemicellulose	[115]
	PtrMYB6		inhibit the accumulation of lignin	[116]
	PtrMYB55	AtMYB55	promote the synthesis of lignin and cellulose	[117]
	PtrMYB74		promote the synthesis of cellulose, lignin, and hemicellulose	[118]
	PtoMYB92	AtMYB85	promotes the accumulation of lignin, but inhibits the hemicellulose synthesis	[119]
	PtrMYB121	AtMYB55	promote the synthesis of lignin and cellulose	[117]
	PtoMYB125	AtMYB85	promotes the accumulation of lignin, but inhibits the hemicellulose synthesis	[119]
	PtrMYB152	AtMYB43	promotes the accumulation of lignin	[120]
	PtoMYB156		inhibit the accumulation of cellulose, lignin, and hemicellulose	[121]
	PtoMYB170	AtMYB61	promotes the accumulation of lignin	[122]
	PtrMYB189		inhibit the accumulation of cellulose, lignin, and hemicellulose	[123]
	PtoMYB216	AtMYB61	promotes the accumulation of lignin	[124]
	PdMYB221		inhibit the accumulation of cellulose, lignin, and hemicellulose	[125]

## Data Availability

The data presented in this study are available on request from the corresponding author. The data are not publicly available due to privacy.
